# Synthesis of Fluorinated Polymers and Evaluation of Wettability

**DOI:** 10.3390/molecules21030358

**Published:** 2016-03-17

**Authors:** Tamami Kimura, Maria Carmelita Kasuya, Kenichi Hatanaka, Koji Matsuoka

**Affiliations:** 1 Institute of Industrial Science, The University of Tokyo, Meguro-ku, Tokyo 153-8505, Japan; s12ds051@mail.saitama-u.ac.jp (T.K.); maria@iis.u-tokyo.ac.jp (M.C.K.); hatanaka@iis.u-tokyo.ac.jp (K.H.); 2 Graduate School of Science and Engineering, Saitama University, Sakura-ku, Saitama 338-8570, Japan

**Keywords:** fluorous, fluorinated polymers, wettability, contact angle, critical surface tension

## Abstract

Two kinds of fluorinated polymers were synthesized: an acrylate polymer having a fluorinated triethylene glycol as a pendant group (**2a**) and a fluoroalkyl acrylate polymer (**2b**). The contact angle of these fluorinated polymers against water, non-fluorinated alcohols and fluorinated alcohols were evaluated. As compared with the fluoroalkyl polymer (**2b**), fluoroethylene glycol polymer (**2a**) showed smaller contact angle against water and non-fluorinated alcohols. This supports the proposition that changing the alkyl chain into the ethylene glycol-type chain gave some interaction between etheric oxygen and water or non-fluorinated alcohols. In addition, fluoroalkyl acrylate polymer (**2b**) showed remarkably low values of critical surface tension.

## 1. Introduction

The regulation of surface property is an important consideration in preparation of materials. Polystyrene is used as hydrophobic materials for Petri dishes to support cell adherence [1]. The high molecular weight poly(ethylene glycol) (PEG) is used to improve hydrophilicity. This results in strong interactions between PEG and water, thus preventing protein adsorption [2].

Fluorinated polymers have attracted attention for the preparation of biomaterials due to their unique properties such as thermal stability, chemical stability, flame retardancy, low dielectric constant, low frictional property and unique surface property [3,4,5,6,7,8,9]. Among these properties, the unique surface behavior of fluorinated compounds can prevent fouling of non-fluorinated compounds and can be used as a biocompatible material which prevents protein adhesion [10]. In general, compatibility is discussed in terms of hydrophilicity or lipophilicity. However, highly fluorinated compounds are neither hydrophobic nor lipophobic, they are fluorophilic [11]. 

One of the means to evaluate compatibility between solid and liquid is by measuring the contact angle. A small contact angle (<90°) corresponds to high compatibility, while a large contact angle (>90°) corresponds to low compatibility [12]. Compatibility is predicted from the surface tension of liquids and the critical surface tension of solids. A liquid can wet a solid if the surface tension is lower than the critical surface tension. The critical surface tension according to Zisman’s approach gives further surface information on the interaction between liquid and the solid surface [13,14,15]. In this study, polymers with a fluorocarbon chain that is suitable for biomaterials were synthesized and the contact angle was determined in order to evaluate compatibility. The critical surface tension was calculated according to Zisman’s approach.

## 2. Results and Discussion

### 2.1. Synthesis of Fluorinated Monomers

**Scheme 1:**
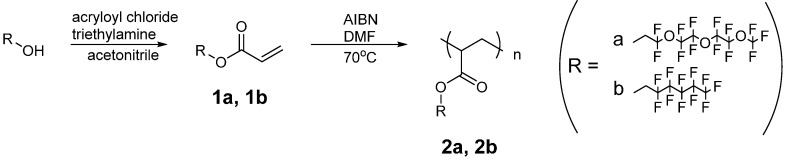
Preparation of fluoroethylene glycol polymer (**2a**) and fluoroalkyl polymer (**2b**).

Two types of monomers were prepared: they are 1*H*,1*H*-perfluoro-3,6,9-trioxadecyl acrylate (fluoroethylene glycol monomer) (**1a**) and 1*H*,1*H*-perfluorohexyl acrylate (fluoroalkyl monomer) (**1b**). The monomers were prepared from 1*H*,1*H*-perfluoro-3,6,9-trioxadecan-1-ol or 1*H*,1*H*-perfluorohexane-1-ol with acryloyl chloride (Scheme 1).

### 2.2. Polymerization of Fluorinated Monomers

Radical polymerization of these monomers using AIBN was carried out to give the corresponding fluorinated polymers. The tacticities of the polymers were confirmed from ^1^H-NMR. The molecular weights of the polymers were calculated by light-scattering spectroscopy and the results are summarized in Table 1. The molecular weight of fluoroalkyl polymer (**2b**) was unexpectedly greater than that of the usually prepared polymer by radical polymerization. The fluoroalkyl polymer (**2b**) was determined to be aggregating. Since a higher than anticipated molecular weight was observed, the fluoroalkyl polymer (**2b**) was hydrolyzed in order to prevent aggregation. After removal of the fluoroalkyl chains from the polymer, the remaining polymer showed the anticipated degree of polymerization.

### 2.3. Contact Angle Measurements

Both polymers were dissolved in Novec™ 7100 (Tokyo, Japan). A silicon wafer surface was coated with polymers by solution casting method. Milli Q (water), pentan-1-ol, hexan-1-ol, heptan-1-ol, 1*H*,1*H*,5*H*-octafluoropentan-1-ol, 1*H*,1*H*-perfluorohexan-1-ol and 1*H*,1*H*,7*H*-dodecafluoroheptan-1-ol were dropped on the silicon wafer covered with each polymer and the contact angle was measured.

Fluoroethylene glycol polymer (**2a**) exhibited a smaller contact angle against water and non-fluorinated alcohols than fluoroalkyl polymer (**2b**) indicating that the fluoroethylene glycol polymer (**2a**) is more hydrophilic and more alcoholphilic (Table 2). The contact angle values of both fluorinated polymers against fluorinated alcohols were nearly 0°. These results indicated that both fluorinated polymers interacted more strongly with fluorinated alcohols than with water or non-fluorinated alcohols.

### 2.4. Calculation of the Critical Surface Tension

**Figure 1:**
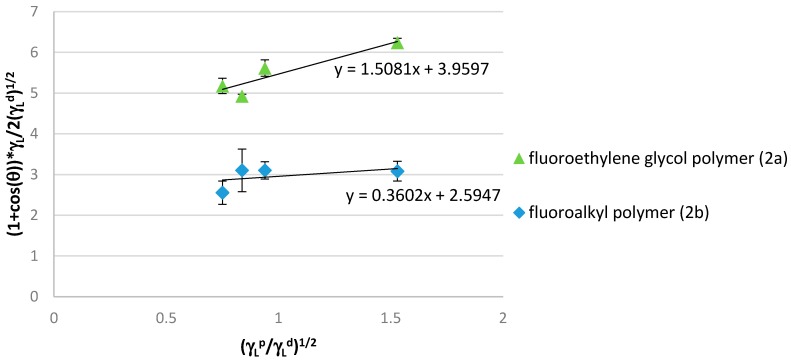
Zisman plots for fluoroethylene glycol polymer (**2a**) and fluoroalkyl polymer (**2b**).

The contact angles against ethylene glycol, propan-1-ol and glycerol were measured to calculate the critical surface tension of each polymer. The calculated results indicated that the fluoroethylene glycol polymer (**2a**) (18 mNm^−1^) has a higher critical surface tension than the fluoroalkyl polymer (**2b**) (7 mNm^−1^) as shown in Figure 1. The critical surface tension of fluoroalkyl polymer (**2b**) is extremely low when compared with previous reports. The critical surface tension of fluoroethylene glycol polymer (**2a**) is similar to polytetrafluoroethylene (PTFE) [16].

From the results of contact angle measurements, the fluoroethylene glycol polymer (**2a**) exhibited higher compatibility than the fluoroalkyl polymer (**2b**) with each solvent. Both fluorinated polymers exhibited fluorophilicity because significantly high compatibility was observed against only fluorinated alcohols. The results obtained from the calculated critical surface tension of fluoroalkyl polymer (**2b**) indicated the large contact angle against water because of the remarkable low critical surface tension. Based on these results, the fluoroalkyl polymer (**2b**) has potential application as a stain-resistant biomaterial against the fouling such as hemoglobin adsorption [17].

Fluoroethylene glycol polymer (**2a**) has a similar critical surface tension to PTFE, which has been widely used in various fields including biomaterial. Since PTFE is produced as a solid powder that is insoluble, it is difficult to coat the material surface with PTFE. On the other hand, fluoroethylene glycol polymer (**2a**) dissolves in fluorinated solvents such as Novec^TM^ 7100. Hence, biomaterials may be coated with the fluoroethylene glycol polymer (**2a**).

## 3. Experimental Section

### 3.1. Preparation

#### 3.1.1. 1*H*,1*H*-Perfluoro-3,6,9-trioxadecyl Acrylate (**1a**)

Fluorinated ethylene glycol monomer (**1a**) was synthesized from 1*H*,1*H*-perfluoro-3,6,9-trioxadecan-1-ol (2.0 g, 5.0 mmol) in anhydrous acetonitrile (9.8 mL) with triethylamine (841.9 μL, 6.0 mmol) and acryloyl chloride (444.0 μL, 5.5 mmol) at 0 °C. The mixture was stirred for 2 h at room temperature. After the complete reaction, water was added and extraction was carried out with ethyl acetate. The reaction mixture was washed with saturated sodium hydrogen carbonate, water and brine, dried over anhydrous magnesium sulfate, and concentrated *in vacuo*. The product was purified by column chromatography (1:20 ethyl acetate-hexane) to afford 1*H*,1*H*-perfluoro-3,6,9-trioxadecyl acrylate (**1a**), 39.8%, as a fluorinated ethylene glycol monomer. ^1^H-NMR (CDCl_3_, 600 MHz): δ 4.53 (t, *J* = 18.66 Hz, 2H), 5.96 (d, *J* = 10.44 Hz, 1H), 6.15 (dd, *J* = 10.44, 17.58 Hz, 1H), 6.49 (d, *J* = 17.58 Hz, 1H); ^13^C-NMR (CD_3_OD, 150 MHz): δ 61.31, 61.53, 61.75, 126.57, 133.13, 164.17.

#### 3.1.2. 1*H*,1*H*-Perfluorohexyl Acrylate (**1b**)

Fluoroalkyl monomer (**1b**) was synthesized from 1*H*,1*H*-perfluorohexan-1-ol (1.9 mL, 10.3 mmol) in anhydrous tetrahydrofuran (20.0 mL) with triethylamine (4.3 mL, 30.9 mmol) and acryloyl chloride (2.5 mL, 30.9 mmol) at 0 °C. The mixture was stirred overnight at room temperature and water was added to the mixture. Extraction was carried out with dichloromethane and the organic layer was washed with saturated sodium hydrogen carbonate, water and brine, dried over anhydrous magnesium sulfate, and concentrated *in vacuo*. The residual mixture was purified by distillation under reduced pressure at 35 °C to yield pure 1*H*,1*H*-perfluorohexyl acrylate (**1b**), 68.0%, as a fluoroalkyl monomer. ^1^H-NMR (CDCl_3_, 600 MHz): δ 4.67 (t, *J* = 26.94 Hz, 2H), 6.00 (d, *J* = 10.50 Hz, 1H), 6.19 (dd, *J* = 10.44, 17.58 Hz, 1H), 6.53 (d, *J* = 17.58 Hz, 1H); ^13^C-NMR (CD_3_OD, 150 MHz): δ 59.39, 59.56, 59.75, 126.57, 133.34, 164.31.

#### 3.1.3. Polymerization of **1a** and **1b**

The fluorinated ethylene glycol (**1a**) and fluoroalkyl (**1b**) monomers were polymerized overnight at 70 °C using α,α′-azobisisobutyronitrile (AIBN) as a free radical initiator in anhydrous *N*,*N*-dimethylformamide (DMF) (321.1 μL). The reaction mixtures were dissolved in Novec™ 7100 and the products were purified by reprecipitation using methanol.

Both fluorinated polymers dissolved in Novec™ 7100 and their molecular weights were calculated by static light scattering spectroscopy, respectively. Fluorinated ethylene glycol polymer (**2a**) (50 mg) and fluoroalkyl polymer (**2b**) (50 mg) in Novec™ 7100 (1.0 mL) were hydrolyzed overnight at room temperature in the presence of aqueous sodium methoxide (22.7 mg, 0.42 mmol), respectively. The molecular weight of each polymer was estimated by size exclusion chromatography after hydrolysis.

*Fluoroethylene glycol polymer* (**2a**); ^1^H-NMR (CDCl_3_, 600 MHz): δ 1.73–1.88 (br, 1H), 2.04–2.19 (br, 1H), 2.47–2.64 (br, 1H), 4.37–4.59 (br, 2H).

*Fluoroalkyl polymer* (**2b**); ^1^H-NMR (CDCl_3_, 600 MHz): δ 1.41–2.00 (br, 1H), 2.00–2.30 (br, 1H), 2.30–2.75 (br, 1H), 4.40–4.80 (br, 2H).

### 3.2. Evaluation of Contact Angle

Contact angles of the fluorinated ethylene glycol polymer (**2a**) and fluoroalkyl polymer (**2b**) were measured. First, the silicon wafer was cleaned by ultrasonication for 10 min in hexane followed by, ethanol and finally, in chloroform at room temperature. The films were prepared by solution casting method 5 *w*% of each solution was prepared. Each solvent (Milli Q, pentan-1-ol, hexan-1-ol, heptan-1-ol, 1*H*,1*H*,5*H*-octafluoropentan-1-ol, 1*H*,1*H*-perfluorohexan-1-ol, 1*H*,1*H*,7*H*-dodecafluoroheptan-1-ol, ethylene glycol, propan-1-ol and glycerol) (5 μL) was dropped on each polymer and the contact angles were directly measured using a microscope. Each reported value was obtained from at least five different measurements and average values were calculated.

## 4. Conclusions

Two fluorinated polymers were synthesized and their contact angles were evaluated against water, non-fluorinated alcohols and fluorinated alcohols. Polymer (**2a**) having pendant-type fluoroethylene glycol chains showed smaller contact angles than those of the fluoroalkyl polymer (**2b**) against water and non-fluorinated alcohols.

## Figures and Tables

**Table 1:** Molecular weight of fluoroethylene glycol polymer (**2a**) and fluoroalkyl polymer (**2b**).

Polymer	Yield (%)	Molecular Weight ^1^ (Da)	Degree of Polymerization (*n*)	
			Before Hydrolysis ^1^	After Hydrolysis ^2^
**2a**	79.0	4.7 × 10^5^	980	850
**2b**	87.2	1.2 × 10^6^	3200	1000

^1^ Calculated by static light scattering; ^2^ Calculated by size exclusion chromatography.

**Table 2:** The contact angles of fluoroethylene glycol polymer (**2a**) and fluoroalkyl polymer (**2b**) against different solvents.

	Milli Q	1-Pentanol	1-Hexanol	1-Heptanol	1*H*,1*H*,5*H*-Octafluoro-1-pentanol	1*H*,1*H*-Perfluoro-1-hexanol	1*H*,1*H*,7*H*-Dodecafluoro-1-heptanol
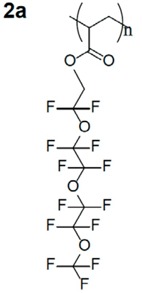	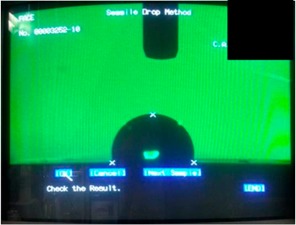	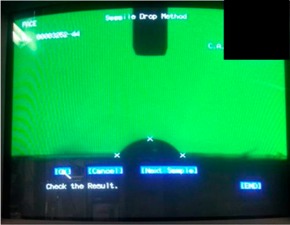	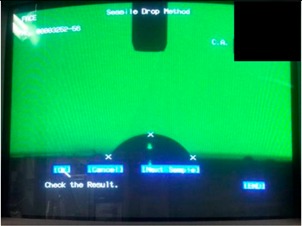	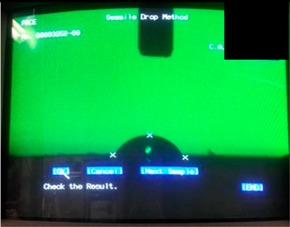	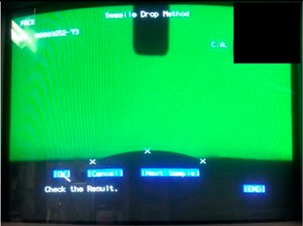	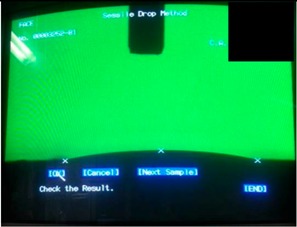	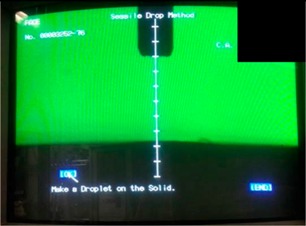
	101.4°	57.4°	58.3°	61.1°	Nearly 0°	Nearly 0°	Nearly 0°
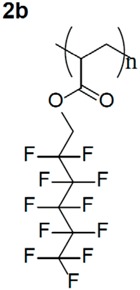	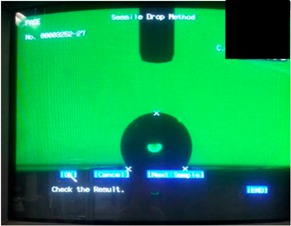	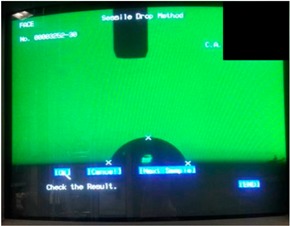	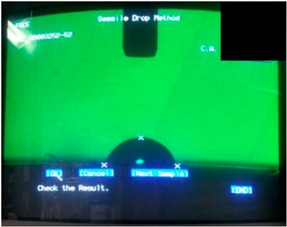	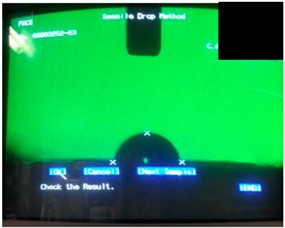	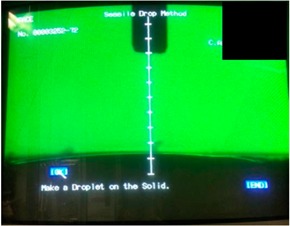	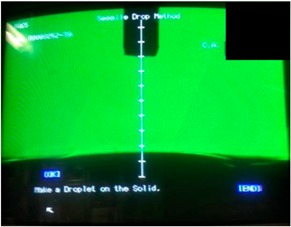	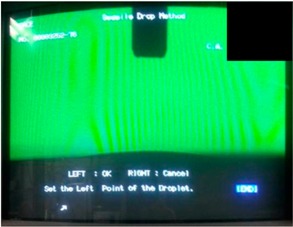
	126.7°	69.1°	78.3°	82.5°	Nearly 0°	Nearly 0°	Nearly 0°

## Abbreviations

The following abbreviations are used in this manuscript:

PEG	poly(ethylene glycol)
PTFE	polytetrafluoroethylene
AIBN	α,α′-Azobisisobutyronitrile
DMF	*N*,*N*-Dimethylformamide
